# Medicinal plants sold in the markets of Antananarivo, Madagascar

**DOI:** 10.1186/s13002-015-0046-y

**Published:** 2015-07-28

**Authors:** Maria Nirina Randriamiharisoa, Alyse R. Kuhlman, Vololoniaina Jeannoda, Harison Rabarison, Nivo Rakotoarivelo, Tabita Randrianarivony, Fortunat Raktoarivony, Armand Randrianasolo, Rainer W. Bussmann

**Affiliations:** Department of Plant Biology and Ecology, Faculty of Science, University of Antananarivo, Antananarivo 101, BP 566 Madagascar; William L. Brown Center, Missouri Botanical Garden, PO Box 299, St. Louis, MO 63166-0299 USA; Missouri Botanical Garden, Madagascar Research and Conservation Program, Antananarivo 101, BP 3391 Madagascar

**Keywords:** Madagascar, Urban market, Medicinal plants

## Abstract

**Background:**

This study focuses on the large outdoor markets of the capital of Madagascar, Antananarivo. As the largest metropolitan area in Madagascar with a population of nearly two million, the region has great capacity for consumption of medicinal plant remedies despite numerous pharmacies. Medicinal plant use spans all socioeconomic levels, and the diverse metropolitan population allows us to study a wide variety of people who consume these plants for medical purposes. The purpose of this study is to identify and generate a list of medicinal plants sold in the traditional markets with a focus on those collected in the forests around Antananarivo, get an idea of the quantities of medicinal plants sold in the markets around Antananarivo, and assess the economy of the medicinal plant markets.

**Methods:**

In order to determine which medicinal plants are most consumed in Antananarivo, ethnobotanical enquiries were conducted in the five main markets of the capital city. Ethnobotanical surveys were conducted with medicinal plant traders, suppliers, harvesters and cultivators, with voucher specimens created from the plants discussed. Trade circuit information was established and the income generated by the trade of some of the species was assessed.

**Results:**

The inventory of the Antananarivo markets resulted in a list of 89 commercialized plant species. Ten of the 89 were mentioned by 60-100 % of vendors. Profitability for vendors is high and competitive with other salaried positions within Antananarivo. Transportation costs are also high and therefore lower profitability for other members in the supply chain.

**Conclusions:**

The markets of Antananarivo have always played a vital cultural role in the lives of urban Malagasy, but our study shows they also play an economic role not only for urban residents but rural harvesters as well. Continued research and monitoring of the non-timber forest products trade in Antananarivo is needed to better understand the impact of trade on the wild plant populations.

## Background

The use of plants for medical treatment and therapy is a practice as old as humanity, dating as far back as the oldest known written documents and found in nearly every known culture [1–3]. Traditional medicine is rich due to the diversity of human groups, languages, and customs, combined with the diversity of ecological regions, leading to innovative plant use and specialized knowledge [4]. The World Health Organization estimates that nearly 80 % of the population in developing countries depends mainly on traditional medicine for the treatment of ailments [5]. The dependence on remedies derived from medicinal plants is particularly important in developing countries where modern medicine is often absent or simply too expensive [6, 7]. Economic devaluation of the developing countries leads to higher prices of pharmaceuticals and makes medicinal plants and traditional medicine more attractive [8]. Additionally, some prefer traditional medicine for various reasons including familiarity, tradition and perceived safety [9, 10].

Medicinal plants can be of great importance in the daily lives of those who live near places where they grow, not only for their healing traditions but as a commodity to take to the urban areas where they are not locally found to be sold in the marketplace [11]. Trade of non-timber forest products (NTFP) has been a mainstay for rural economies with a large majority being sourced from wild populations [12]. Rural farmers and residents therefore have a financial interest to not only exploit and develop trade of these natural resources [13], but also to consider conservation measures [14, 15]. The domestic market of medicinal plants of Madagascar is not well documented, and the market for medicinal plants and derivatives only represents a small fraction compared to all internal and external trade of the country [16]. Our study focused on the city of Antananarivo and its medicinal plant markets. As the capital of Madagascar Antananarivo is the largest metropolitan area with a population of nearly 2 million, and the region has great potential for consumption of medicinal plant remedies despite numerous allopathic pharmacies [11]. Medicinal plant-use in Madagascar spans all socioeconomic levels and the diverse metropolitan population allowed to study a wide variety of people using plant products. The objective of this study was to identify and generate a list of medicinal plants sold in the traditional markets with a focus on those collected in the forests around Antananarivo, as well as getting information on the quantities of medicinal plants sold in the markets around Antananarivo, and to assess the economy of the medicinal plant markets. Interviews were started with the vendors at the major markets of Antananarivo, and continued with suppliers wherever possible. We then tried to elucidate who cultivated or harvested plants sourced by the suppliers and finally who held the knowledge of traditional plant medicine for the region.

## Methods

### Study area

Antananarivo is the capital of Madagascar, the fourth largest island in the world, and centrally located in the highlands at nearly 1,300 meters above sea level [17]. We conducted surveys in five major markets of Antananarivo: the Esplanade Analakely, Petite Vitesse, Pavilion Analakely, Isotry and Andravohangy. These markets were chosen based on the following criteria: market size and popularity, medicinal plant species sold on the premises, and knowledge of vendors regarding the use and sale of medicinal plants. Furthermore, markets in Antananarivo are housed in permanent buildings where vendors occupy permanent booths, which allowed for repeat visits to the same vendor to update lists and conduct further interviews.

### Markets

The medicinal plant market includes two subsectors: the traditional medicinal plant market and the pharmaceutical market. The traditional plant market, known as *raokandro*, includes plants for public use with little to no processing (dried, raw material). The plants were sold either singularly or as a mix with other plants for a particular treatment. Other types of legal plant markets in Antananarivo are pharmaceutical, cosmetics and aromatherapy shops marked with HOMEOPHARMA and IRMA, selling mostly medicinal plants and medicinal plant products that have undergone extensive modification (liquid extract, cream, ointment). The present study focused on the medicinal plant trade within the raokandro. A variety of actors were involved in the sale of medicinal plants. These included operators, collectors, harvesters, and small retailers. The definitions we followed were taken from the ministerial decree number 2915/87 of 30 June 1987 and the Decree of 17 November 1930 mentioned in Articles 32 and 33 are presented in Table 1.

**Table 1:** Definition of participants within the herbal market trade scheme. Types of collectors and their role within the trade as defined by the Madagascar government

Operators	Persons who legally hold a license or an operating agreement to operate and collect medicinal plants and forest products to sell or use as raw materials.
Collectors	These are individuals who collect plants from those who harvest in the forest. They are authorized to carry out the grouping of plants with several collectors.
Harvesters	These are the persons authorized to conduct harvesting or gathering medicinal plants for commercial purposes
Rural harvesters	Those who come from the rural areas surrounding the city of Antananarivo to deliver medicinal species to the market sellers
Urban harvesters	people living in the vicinity of the capital, which also make deliveries to vendors of medicinal plants in the traditional market of Antananarivo
Public resellers (vendors)	These are the people who sell plants to the public. Called "tapa-mpivarotra kazo" or "mpivarotra raokandro” in Malagasy.

### Ethnobotanical surveys

To gather information about the market of medicinal plants, a series of semi-structured interviews were conducted with traders at the traditional markets (*raokandro)* of Antananarivo after obtaining oral prior informed consent. Questionnaires were used as a foundation for discussions with the collectors and traders. During market interviews we conducted our survey individually and iteratively [18]. All medicinal species that were discussed with the vendors were also purchased from the vendors at the regular price. Medicinal plants were then identified at the department of Plant Biology and Ecology at the University of Antananarivo and crosschecked with published ethnobotanical and floristic literature where available [19–22]. Plant names follow www. TROPICOS.org. Herbarium vouchers were deposited at the herbaria of Centre National de la Recherche Appliquée au Developement Rural (TEF), Parc de Tsimbazaza (TAN) and Missouri Botanical Garden (MO).

### Statistical analysis

For each medicinal plant a Use Index (UI%) was calculated to give a ranking of the importance of the use and trade of medicinal species in markets of Antananarivo. The UI% is calculated from the formula, UI = (na/NA) x 100, where na is the number of interviewees who cite the species as useful and NA is the totally number of people interviewed [23]. In this case, na represents represent the number of vendors who sell a particular medicinal species. The following formulas were used to calculate the profit margin of the various intermediaries surveyed. For sellers, Bv = PV- PA where the benefit to vendors (Bv) is the difference between the sale price (PV) and the purchase price (PA). For harvesters (rural and urban), Bh = ΣR - ΣEx, where the benefit to harvesters (Bh) is the difference between the revenue (R) and expenditure costs (Ex). Profit margin (PM) was calculated with PM = B / ΣR, based on [23].

## Results and discussion

We interviewed 86 people in the traditional markets of medicinal plants in Antananarivo. Table 2 summarizes the survey sites and the number of informants surveyed.

**Table 2:** Market sites and number of informants surveyed

Market	Number of vendors	Rural harvesters	Intermediaries or Urban harvesters
Esplanade Analakely	9	0	3
Petite Vitesse	21	15	7
Andravoahangy	21	5	0
Pavilion Analakely	2	0	0
Isotry	3	0	0
Total	56	20	10
Total interviewed	86		

We were able to identify 89 medicinal plant species from 56 vendors. A list of medicinal plants is presented in Table 3. The actual number of species sold is likely higher than what we were able to identify because of the study’s limited duration [24]. Furthermore, vendors spoke only about plants that at the time of the interview were available in their stalls. Other plants might be sold at other times, but if they were not available for purchase the sellers did not mention them.

**Table 3:** List of medicinal plants sold at the Antananarivo medicinal markets. Scientific name, vernacular name, plant part used, disease treated and voucher number [MTR = Randriamiharisoa, Maria T.] for all 89 plants identified at the Antananarivo Markets. Use citations were compared with Madagascar ethnobotany published literature: [1] Boiteau P, Allorge- Boiteau L, 1993; [2] Samyn, JM, 1999; [3] Gurib-Fakim A, Brendler T, 2004

Scientific name	Vernacular name	Part used	Application	Uses cited in literature	Voucher number
Acanthaceae					
*Avicennia marina* (Forssk.) Vierh*.*	Afiafy	Leaf	Stomach ulcer		MTR142
*Justicia* sp*.*	Belohalika	Leaf	Neuralgia		MTR190
Amaranthaceae					
*Cyathula uncinulata* (Schrad.) Schinz	Tangogo	Leaf	Stomach ulcer, hepatitis, diabetes, cardiac problems		MTR163
Anacardiaceae					
*Anacardium occidentale* L.	Mahabibo	Leaf	Diabetes, hemorrhoids, stomach ulcer, allergies, hepatitis, wounds, incontinence, anorexia		MTR127
*Rhus taratana* (Baker) H. Perrier	Andriambavimahery	Leaf	Wounds, stomach ulcer		MTR174
Apiaceae					
*Centella asiatica* (L.) Urb.	Talapetraka	Entire plant	Stomach ulcer, wounds	Wounds^3^, skin eczema^3^, accesses^3^, conjunctivitis^3^	MTR138
Apocynaceae					
*Catharanthus lanceus* (Bojer ex A. DC.) Pichon	Vonenina	Root	Cancer	Diuretic^2^, purgative^2^, vermifuge^2^, sores^2^	MTR161
*Catharanthus roseus* (L.) G. Don	Vonenina	Root	Cancer, appetite suppressant	Hypotensive^1^, antidepressant^1^, antitumoral^1^, purgative^2^, diabetes^2^, appetite suppressant^2^, vermifuge^3^, diarrhea^3^, dysentery^3^	MTR162
*Cynanchum* sp.	Vahamavo	Leaf	Asthenia, erectile dysfunction		MTR191
*Pentopetia* sp.	Tandrokosy	Leaf	Cough, hepatitis, neuralgia		MTR189
Araliaceae					
*Schefflera bojeri* (Seem.) R. Vig.	Tsingila	Leaf	Stomach ulcer, hepatitis		MTR143
*Schefflera* sp.	Ramadio	Leaf	Neurasthenia, back pain		MTR144
Asteraceae					
*Brachylaena ramiflora* (DC.) Humbert	Ramanjavona	Leaf	Asthenia, stomach ulcer,		MTR173
*Cynara scolymus* L.	Artichaut	Leaf	Stomach ulcer, hepatitis		MTR192
*Distephanus polygalifolius* (Less.) H. Rob. & B. Kahn	Ninginingina	Leaf	Syphilis, neuralgia, back pain, stomach ulcerm, hepatitis, albumin, incontinence		MTR136
*Emilia citrina* DC.	Tsiotsiona	Leaf	Asthenia, anorexia		MTR202
*Helichrysum faradifani* Scott- Elliot	Haihalala	Leaf	Gonorrhea, cough, asthenia, fever, stomach ulcer, hepatitis		MTR159
*Helichrysum gymnocephalum* (DC.) Humbert	Rambiazina	Leaf	Stomach ulcer, cough, wound, severe headache	Headaches^1^, bronchitis^1^, ulcers^1^, heartburn^2^, upset stomach^2^, fever^2^, diarrhea^3^, dysmenorrhea^3^, rheumatism^3^, gout^3^	MTR160
*Inulanthera brownii* (Hochr.) Källersjö	Kelimavitrika	Leaf	Immune system of children, erectile dysfunction, stiffness		MTR128
*Psiadia altissima* (DC.) Drake	Sakatavilotra	Leaf	Cough, wound, diarrhea	Fever^3^, abdominal pain^3^, antiseptic^3^, toothache^3^, boils^3^	MTR220
*Senecio canaliculatus* Bojer ex DC.	Ramijaingy	Leaf	Stomach ulcer, gastroenteritis, syphilis		MTR201
*Vernonia appendiculata* Less.	Ambiaty	Leaf	Fever, nerves		MTR193
Bignoniaceae					
*Jacaranda mimosifolia* D. Don	Zaharandaha	Leaf	Sinusitis, severe headache		MTR145
*Phyllarthron bojeranum* DC.	Zahana	Leaf	Asthenia, erectile dysfunction, severe headache, gonorrhea, cough, syphilis		MTR175
*Symphytum orientale* L.	Konsody ou Maseza	Leaf	Stomach ulcer, hepatitis		MTR203
Cactaceae					
*Cereus triangularis* (L.) Haw.	Tsilo	Root	Kidney stones, urinary tract problems, syphilis, gonorrhea		MTR158
Canellaceae					
*Cinnamosma madagascariensis* Danguy	Mandravasarotra	Bark	Astenia, erectile dysfunction, stomach ulcer	Stomach pain^3^, colic^3^, analgesic^3^, indigestion^3^, stimulant^3^, cough^3^, dysentery^3^	MTR194
Celastraceae					
*Mystroxylon aethiopicum* (Thunb.) Loes.	Fanazava	Leaf	Neuralgia, hepatitis, albumin, erectile dysfunction, back pain, urinary tract problems, stomach ulcer, hypertension, immune deficiency	Fatigue^3^,neuralgia^3^, purgative^3^, vertigo^3^	MTR126
Combretaceae					
*Combretum coccineum* (Sonn.) Lam.	Tamenaka	Fruit	Intestinal parasites	Anthelmintic^,3^, liver problems^3^	MTR200
*Terminalia catappa* L.	Atafana	Leaf	Urinary tract problems	Astringent^3^, sudorific^3^, dysentery^3^	MTR188
Commelinaceae					
*Commelina madagascarica* C.B. Clarke	Nifinakanga	Leaf	Abortifacient, acne		MTR176
Crassulaceae					
*Kalanchoe prolifera* R. Hamet	Sodifafana	Leaf	Neurasthenia	Boils^3^, furuncles^3^, wounds^3^, rheumatism^3^	MTR186
Cyperaceae					
*Cyperus papyrus* subsp. *madagascariensis* (Willd.) Kük.	Fonjozoro	Stem	Emphysema, back pain		MTR146
Droseraceae					
*Drosera madagascariensis* DC.	Mahantanando	Leaf	Conjunctivitis, enurensis	Coughs^3^, toothpaste^3^, dyspepsia^3^, anemia^3^	MTR129
Ebenaceae					
*Diospyros* sp.	Bois de rose	Bark	Cysticercosis, intestinal parasites, taxoplasmosis, emphysema, diabetes, albumin regulation, allergies		MTR171
Equisetaceae					
*Equisetum* sp.	Tsitoatoana	Leaf	Constipation, urinary tract problems		MTR177
Euphorbiaceae					
*Ricinus communis* L.	Tanantanamanga	Leaf	Asthenia, hemorrhoids, wounds, intestinal parasites, cold	Galactagogue^1,2^, purgative^1,2^, laxative^1,2^, intestinal worms^1^, tapeworm^1^, headache^2^, rheumatism^2^, dental cavities^2^, wounds^2^, fevers^2^	MTR164
Fabaceae					
*Caesalpinia bonduc* (L.) Roxb.	Vatolalaka	Fruit	Hemorrhoids, appendicitis		MTR204
*Phylloxylon xylophylloides* (Baker) Du Puy, Labat & Schrire	Arahara	Leaf	Hepatitis, urinary tract problems, pharyngitis		MTR184
*Senna septentrionalis* (Viv.) H.S. Irwin & Barneby	Anjanajana	Leaf	Immune system children, gastroenteritis		MTR147
*Senna occidentalis* (L.) Link	Tsotsorinangatra	Stem	Syphilis, gonorrhea, prostate tumor, hypertension, hepatitis, rheumatism		MTR165
*Tamarindus indica* L.	Voamadilo	Leaf	Constipation, gastroenteritis, wounds	Laxative^1^,vermifuge^1^, stomach ache^1^, general wounds^1^	MTR125
Gentianaceae					
*Tachiadenus longifolius* Scott- Elliot	Tapabatana	Leaf	Diarrhea, stomach ulcer		MTR172
Gesneriaceae					
*Streptocarpus hilsenbergii* R. Br.	Mangavony	Enitre plant	Hepatitis, acne		MTR185
Hydrostachyaceae					
*Hydrostachys stolonifera* Baker	Tsilavondrina	Leaf	Asthenia		MTR187
Hypericaceae					
*Harungana madagascariensis* Lam. ex Poir.	Harongana	Leaf	Wounds, asthma, cough, stomach ulcer, hepatitis, gastroenteritis, albumin, allergies, insomnia	Scabies^1,2^, stomach ache^1^, flatulence^1^, anticatarrhal^1,2^, bladder infections^2^, syphilis^2^, menstruation regulation^2^, fever^2^, wounds^2^, diarrhea^2,3^, hemorrhoids^2^, skin diseases^3^	MTR130
*Psorospermum* sp.	Todihazo	Stem	Scabies, leprosy		MTR148
*Psorospermum ferrovestitum* Baker	Andriambolamena	Leaf	Female infertility, abortifacient, stomach ulcer, hypertension, intestinal parasites		MTR166
Lamiaceae					
*Ocimum gratissimum* L.	Romba	Leaf	Severe headache, albumin, wounds, abortifacient, cold, low calcium, dental problems	Digestion^3^, chest complaints^3^, diarrhea^3^, vomiting^3^, anticatarrh^3^, antiseptic^3^	MTR205
*Tetradenia riparia* (Hochst.) Codd	Borona	Leaf	Cough, wounds, hepatitis		MTR221
Lauraceae					
*Cinnamomum camphora* (L.) J. Presl	Ravitsara	Leaf	Stomach ulcer, hepatitis, abortifacient, jaundice, hypertension, appendicitis, rheumatism	Fevers^3^, rheumatism^3^, abortifacient^3^	MTR122
Loganiaceae					
*Anthocleista madagascariensis* Baker	Landemy	Leaf	Stomach ulcer, diarrhea, malaria, constipation, abdominal colic, severe headache	Fever^1,2^, dysentery^1,2^, emetic^1,2^, laxative^1,2^	MTR149
Lycopodiaceae					
*Lycopodium* sp.	Karakaratoloha	Leaf	Hepatitis, hypertension, gastroenteritis, epilepsy		MTR157
Meliaceae					
*Azadirachta indica* A. Juss.	Nimo	Leaf	Asthenia, diabetes, albumin, rheumatism, pelvic pain, boils, hepatitis, kidney stones, burns, constipation, high cholesterol		MTR124
*Cedrelopsis grevei* Baill.	Katrafay	Bark	Asthenia, erectile dysfunction, neurasthenia, back pain		MTR141
*Neobeguea mahafaliensis* J.-F. Leroy	Andy	Bark	Asthenia, erectile dysfunction'		MTR183
Molluginaceae					
*Mollugo nudicaulis* Lam.	Aferotany	Entire plant	Cough, gastroenteritis		MTR178
Moraceae					
*Ficus reflexa* Thunb.	Nonoka	Leaf	Hepatitis, gastroenteritis, wounds, albumin, hemorrhoids		MTR167
*Morus alba* L.	Voaroihazo	Leaf	Low calium, anorexia		MTR209
Primulaceae					
*Embelia concinna* Baker	Tanterakala	Leaf	Intestinal parasites, erectile dysfunction		MTR206
Myrtaceae					
*Eucalyptus citriodora* Hook.	Kininina oliva	Leaf	Cold, severe headache		MTR210
*Eucalyptus* sp.	Kininimpotsy	Leaf	Cold, severe headache		MTR211
*Syzygium cumini* (L.) Skeels	Rotra	Bark	Diarrhea, gastroenteritis		MTR131
Nymphaeaceae					
*Nymphaea* sp.	Betsimilana	Leaf	Female infertility, abortifacient, albumin, painful menstruation		MTR219
Onagraceae					
*Ludwigia octovalvis* (Jacq.) P.H. Raven	Volondrano	Leaf	Emphysema	Nose bleeds^3^, diarrhea^3^, malnourishment^3^	MTR150
Orchiaceae					
*Vanilla madagascariensis* Rolfe	Vahinamalona	Stem	Erectile dysfunction, asthenia	Aphrodisiac^1^,	MTR208
Pedaliaceae					
*Uncarina* sp.	Farehitra	Leaf	Acne	Dandruff^3^, alopecia^3^	MTR132
Poaceae					
*Cynodon dactylon* (L.) Pers.	Fandrotrarana	Entire plant	Syphilis, kidney stones		MTR168
*Imperata cylindrica* (L.) Raeusch.	Fakatenina	Root	Kidney stones		MTR182
*Zea mays* L.	Volokatsaka	Silk	Urinary tract problems, hepatitis, kidney stones		MTR156
Pteridaceae					
*Adiantum capillus-veneris* L.	Ampanga	Leaf	Allergies, cough	Respiratory problems^1^, diuretic^1^, chickenpox^1^, measles^1^	MTR207
Ranunculaceae					
*Clematis mauritiana* Lam.	Farimafy	Leaf	Stomach ulcer, hepatitis, erectile dysfunction	Antiasthmatic^3^, rheumatism^3^, cough^3^, bronchitis^3^, abdominal pains^3^	MTR179
Rubiaceae					
*Oldenlandia* sp.	Ahipody	Leaf	Scabies, leprosy		MTR218
*Paederia foetida* L.	Vahamaibo, laingomaimbo	Leaf	Dental issues, wound, stomach ulcer, gastroenteritis	Diuretic^1,3^, diaphoretic^1^, purgative^1^, skin issues^1,3^, ulcers^1,^ boils^3^, venereal diseases^3^, bladder issues^3^, gastric pains^3^	MTR123
*Pauridiantha paucinervis* (Hiern) Bremek.	Tamirova	Leaf	Stomach ulcer, hepatitis, hypertension, urinary tract problems, rheumatism, malaria, albumin, diabetes		MTR153
Rutaceae					
*Toddalia asiatica* (L.) Lam.	Fanala simba	Elaf	Syphilis, gonorrhea	Malaria^3^, digestive complaints^3^, fever^3^, cholera^3^, diarrhea^3^, rheumatism^3^, syphilis^3^	MTR181
Salicaceae					
*Homalium parkeri* Baker	Hazomby	Bark	Dental issues		MTR140
Salviniaceae					
*Azolla* sp.	Ramilamina	Lower	Cardiac arrest		MTR170
Smilacaceae					
*Smilax anceps* Willd.	Avotra	Leaf	Gastroenteritis, abdominal colic	Varicose veins^3^,eczema^3^, liver disorders^3^	MTR180
Solanaceae					
*Brugmansia candida* Pers.	Datroa	Leaf	Epilepsy, paraplegia		MTR152
*Physalis peruviana* L.	Voanantsindrana	Leaf	Rheumatism, urinary tract problems, syphilis, stomach ulcer, hepatitis	Eat berries before physical exertion^1^, diuretic^1,3^, kidney stones^1^, rheumatism^1^, abscess^2^, liver disease^2^, gout^3^, fever^3^, heart palpitations^3^, emollient^3^	MTR137
*Solanum mauritianum* Scop.	Seva	Leaf	Hepatitis, wound	General disinfectant^1^, Stomach ulcers^2^	MTR151
Stilbaceae					
*Nuxia capitata* Baker	Valanirana	Leaf	Gastroenteritis, asthenia, cough		MTR169
Urticaceae					
*Urera acuminata* (Poir.) Gaudich. ex Decne.	Sampy vato	Leaf	Kidney stones, abortifacient, hepatitis, stomach ulcer	Irritant to skin and eyes^3^, childbirth^3^	MTR133
Verbenaceae					
*Lantana camara* L.	Randriaka	Leaf	Hemorrhage, hypertension		MTR155
Xanthorrhoeaceae					
*Aloe macroclada* Baker	Vahona	Leaf	Cancer, allergies, acne, fungus		MTR139
*Dianella ensifolia* (L.) DC.	Erana	Leaf	Intestinal parasites, constipation, back pain, gonorrhea	Eczema^3^, dysentery^3^, stomach pains^3^	MTR154
Zingiberaceae					
*Zingiber* sp.	Tamotamo	Tuber	Cough		MTR135
*Zingiber officinale* Roscoe	Sakamalao	Tuber	Cough		MTR134

**Fig. 1:**
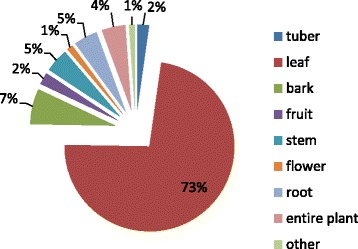
Plant parts most commonly sold

**Fig. 2:**
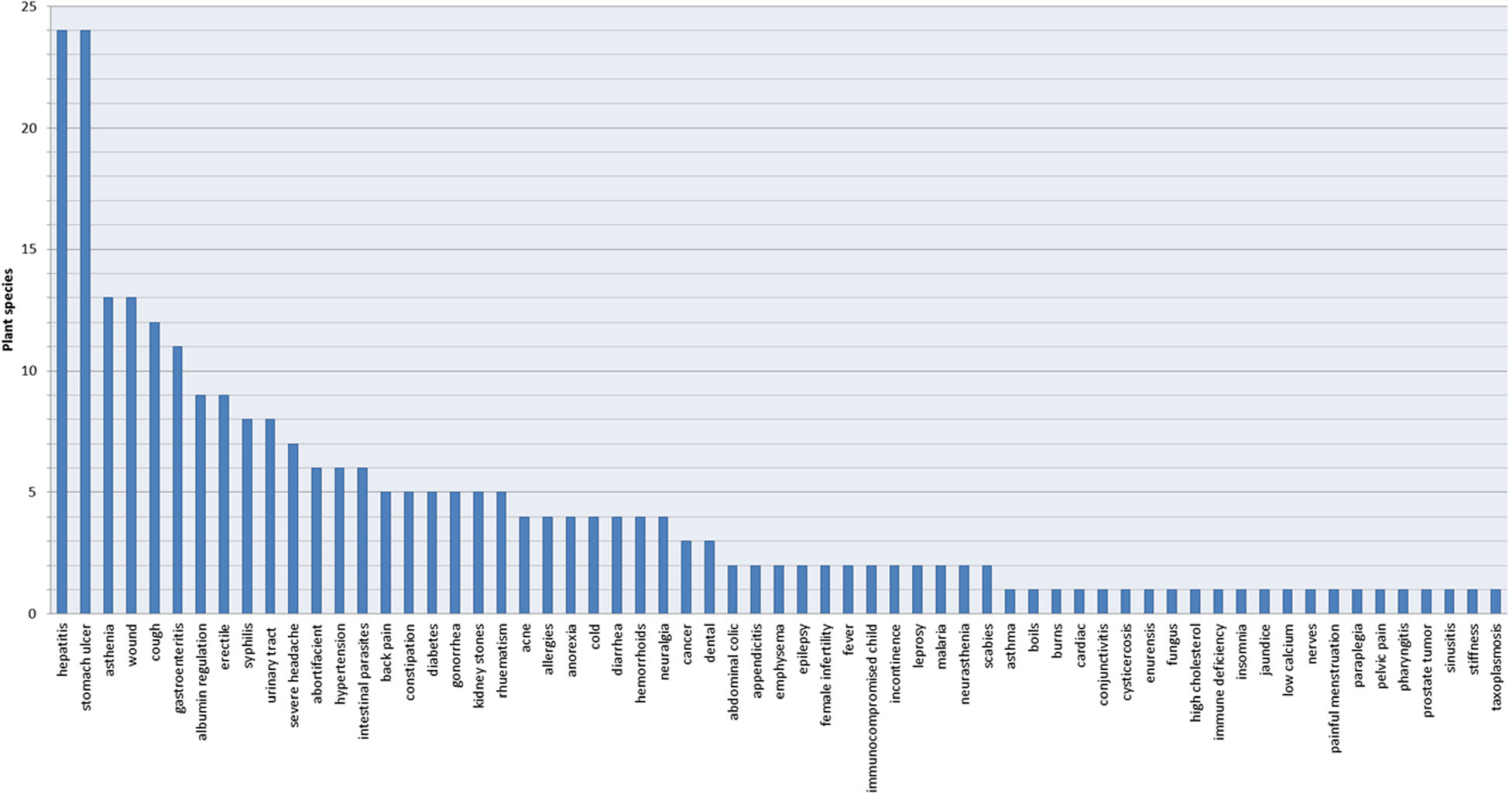
Number of plant species sold for specific ailments

Among the medicinal species available at the major markets of the city of Antananarivo, we encountered nine plant part used: leaves (73 %), bark (7 %), stems (5 %), roots (5 %), entire plant (4 %), fruit (2 %), tuber (2 %), flower (1 %), other (1 %). (Fig. 1) Leaves were by far the most common plant material used, followed by bark. While leaves and bark were often well represented in other studies, only 50 % of the combined total in our study were leaves and bark, similar to in Sierra Leone [25]. These most common health complaints treated with plants were hepatitis, kidney stones, asthenia, wounds, coughs and gastroenteritis (Fig. 2).

### Most traded medicinal species

**Fig. 3:**
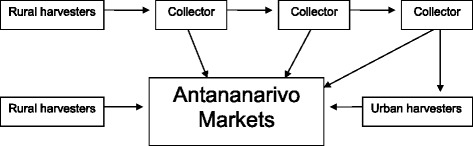
Market chain of medicinal plants in sold in Antananarivo

Table 4 lists the ten most traded species in the markets, including the Use Index calculated for each of these species, which varied from 61 % to 100 %. Prices are typically the main economic indicators about the supply and demand for a product, with higher prices indicating species with higher demand and lower supply. However, we found that the organization of economic actors within the regional medicinal plant trade was also a determinant of prices, often affecting the price based on who and how the species was sourced. Vendors bought their plants from rural harvesters, urban harvesters, and collectors, which is a common trade pattern found in other parts of Africa as well [26]. Increased number of intermediaries before a species reaches the sellers increased the price on the market. Two commercial channels could be distinguished: a short circuit, when harvesters moved to Antananarivo to be closer to the markets in order to sell their products directly themselves, and a long circuit, consisting of a long chain of intermediaries the products passed through before reaching sellers in Antananarivo (Fig. 3). The purchase price of medicinal plants varied widely depending on the species, but we found that prices were constant for a given species.

**Table 4:** Use index calculated for the most traded species and their treatment associations

Family	Scientific name	Vernacular name	Application	Use index
Rubiaceae	*Pauridiantha paucinervis* (Hiern) Bremek.	Tamirova	Stomach ulcer, hepatitis, high blood pressure, urogenital diseases, rheumatism, malaria, edema, diabetes	100 %
Meliaceae	*Cedrelopsis grevei* Baill.	Katrafay	Asthenia, erectile dysfunction, back pain	100 %
Meliaceae	*Neobeguea mahafaliensis* J.-F. Leroy	Andy	Asthenia, erectile dysfunction	82 %
Cactaceae	*Cereus triangularis* (L.) Haw.	Tsilo	Kidney stones, dysuria, anuria, syphilis, gonorrhea	78 %
Fabaceae	*Senna occidentalis* (L.) Link	Tsotsorinangatra	Syphilis, gonorrhea, enlarged prostate, high blood pressure, rheumatism, hepatitis	70 %
Lamiaceae	*Ocimum gratissimum* L.	Romba	Intense headache, edema, wounds, repeated miscarriages, cold, hypocalcemia, dental pain	65 %
Boraginaceae	*Symphytum orientale* L.	Konsody	Stomach ulcer, hepatitis	65 %
Asteraceae	*Cynara cardunculus* subsp. *flavescens* Wiklund	Artichaut	Stomach ulcer, hepatitis	64 %
Asteraceae	*Distephanus polygalifolius* (Less.) H. Rob. & B. Kahn	Ninginingina	Syphilis, neuralgia, back pain, stomach ulcer, hepatitis, edema, enuresis	61 %
Urticaceae	*Urera acuminata (Poir.) Gaudich. ex Decne.*	Sampivato	Kidney stones, repeated miscarriages, hepatitis, stomach ulcer	61 %

However, product price increased with each change of hands as transportation costs or other fees incurred. As found in other parts of the world, the amount of time, energy and resources needed to transport medicinal plants to the market was considered extremely high [27]. In addition, the price also fluctuated depending on the customer's apparent wealth and the type of market (i.e.: tourist handicraft market). Medicinal plants were often supplied from a collector two to four times a week, while some species were only delivered once a month or once a year (in the case of plants came from other provinces of Madagascar). Urban harvesters could afford to bring small amounts of plants (a basket or box) as they sold their products almost daily. Table 5 summarizes the types of providers and delivery frequency by type of market.

**Table 5:** Suppliers and frequency of deliveries at each market site

Market	Frequency of delivery			Transportation
	Rural harvesters	Urban harvesters	Collection	
Andravoahangy	2 times a week	Daily	3x/month	By foot
Isotry	Irregular	Daily	Irregular	By foot
Petite vitesse	4 times a week	Daily	1- 2 / week	By foot
Esplanade Analakely	Irregular	Daily	Irregular	By foot

Local markets worldwide are a thriving business for both rural and urban dwellers, with a steady demand for medicinal plants. To understand the possible benefits for rural harvesters, several factors needed to be taken into account: 1) the cost of transporting goods 2) the frequency of deliveries to the Antananarivo markets 3) the quantity and value of the species transported to the market. Transport costs from rural areas of Antananarivo depended greatly upon the state of the road and mode of transportation and varied from $ 0.45 - $ 1.34 per person transporting plants. The most common mode of transport was carrying plant products “on their backs”, or by hand, from the rural areas to the city market, with costs ranging from $ 0.08 – $ 0.15 per bag. Overall, transportation costs to deliver the goods to the vendors of medicinal plants in the major markets of the city of Antananarivo ranged anywhere from $ 3.39 - $ 8.57 per week. If four bags of medicinal plants (which was the standard weekly amount per vender) were sold at a price of $ 4 - $ 5 per bag, earnings were $ 12 - $ 20 a week. The profit margin ranged from 40 % - 81 %.

### Case study: *Pauridiantha paucinervis* and *Mystroxylon aethiopicium*

To further analyze the trade value of the medicinal plants in Antananarivo, we used the most used single species, *Pauridiantha paucinevris*, and a species that was present in most of the mixtures, *Mystroxylon aethiopicium* for closer analysis*.*

In the market, *Pauridiantha paucinervis* was sold packaged in a sealed, labeled bags. We found that package was uniform in all markets. Collectors sold this product to vendors for an average of $ 0.06 per package, and the frequency of deliveries was based on fluctuating demand in the markets. The selling price of the product in the market ranged from $ 0.08 - $ 0.17. Thus, the selling price of this product was double or even triple compared to its purchase price. According to our surveys vendors sold an average of six bags of *P. paucinervis* each day. Thus, the average earnings for the sale of *P. paucinervis* amounted to $ 0.50 per day, and the monthly earnings could be upwards of $ 22.50.

*Mystroxylon aethiopicium* was sold at $ 0.10 - $ 0.20 per package, but this species was only rarely sold alone, but rather was packaged with other herbs to form a tea to treat specific ailments. Sellers bought from collectors once a week, and the order quantity, depending heavily on supply and demand, was often irregular. The purchase price of this species from suppliers was $ 0.03 – $ 0.30, depending on volume. The profit margin of sales was 100 % to 150 % if the plant was sold alone, and even higher if it was combined with other herbs. In the latter case, the sale price varied according to the type of disease and also the amount needed for treatment. Vendors sold an average of 10 packets of *M. aethiopicium* a day, yielding an average of $ 0.30. The average monthly income for a vendor selling *M. aethiopicium* was about $10. Therefore, the combined sale of only *P. paucinervis* and *M. aethiopicium* averaged a monthly gross income of $25. Considering that the professional monthly minimum wage guarantee in Madagascar is $25, the medicinal plant trade can be considered lucrative. However, given the limited amount of time, and limited number of interviews, we could not elucidate the exact quantity of plant material sold in the markets.

## Conclusions

Market studies of non-timber forest products (NFTP) have in the past focused mostly on rural economies and export markets. Recently, increased interest in the domestic marketplace has resulted in more data about economic value of NFTP in the domestic medicinal plant trade. It is difficult to quantify the number of medicinal plants that circulate in the markets of a city like Antananarivo, because this number is highly dependent on market dynamics, which can be quite irregular even for a single plant species. But our estimates show that the sale of medicinal plants in the domestic market provided income for all players - vendors, collectors and harvesters - allowing them to supplement or fully supply their annual income. The impact of these urban traditional markets on the urban and rural economy can be substantial [28]. This booming business has real implications for conservation concerns, which should be researched further to fully explore the impact of the medicinal plant trade on the ecological well-being of the forests where the plants are sourced. Further research and monitoring of the Antananarivo markets will also be invaluable to chart the sustainable use of wild natural resources.
